# Limited Transcriptional Responses of *Rickettsia rickettsii* Exposed to Environmental Stimuli

**DOI:** 10.1371/journal.pone.0005612

**Published:** 2009-05-19

**Authors:** Damon W. Ellison, Tina R. Clark, Daniel E. Sturdevant, Kimmo Virtaneva, Ted Hackstadt

**Affiliations:** 1 Laboratory of Intracellular Parasites, Rocky Mountain Laboratories, National Institute of Allergy and Infections Diseases, National Institutes of Health, Hamilton, Montana, United States of America; 2 Genomics Unit, Research Technology Section, Rocky Mountain Laboratories, National Institute of Allergy and Infections Diseases, National Institutes of Health, Hamilton, Montana, United States of America; Baylor College of Medicine, United States of America

## Abstract

Rickettsiae are strict obligate intracellular pathogens that alternate between arthropod and mammalian hosts in a zoonotic cycle. Typically, pathogenic bacteria that cycle between environmental sources and mammalian hosts adapt to the respective environments by coordinately regulating gene expression such that genes essential for survival and virulence are expressed only upon infection of mammals. Temperature is a common environmental signal for upregulation of virulence gene expression although other factors may also play a role. We examined the transcriptional responses of *Rickettsia rickettsii*, the agent of Rocky Mountain spotted fever, to a variety of environmental signals expected to be encountered during its life cycle. *R. rickettsii* exposed to differences in growth temperature (25°C vs. 37°C), iron limitation, and host cell species displayed nominal changes in gene expression under any of these conditions with only 0, 5, or 7 genes, respectively, changing more than 3-fold in expression levels. *R. rickettsii* is not totally devoid of ability to respond to temperature shifts as cold shock (37°C vs. 4°C) induced a change greater than 3-fold in up to 56 genes. Rickettsiae continuously occupy a relatively stable environment which is the cytosol of eukaryotic cells. Because of their obligate intracellular character, rickettsiae are believed to be undergoing reductive evolution to a minimal genome. We propose that their relatively constant environmental niche has led to a minimal requirement for *R. rickettsii* to respond to environmental changes with a consequent deletion of non-essential transcriptional response regulators. A minimal number of predicted transcriptional regulators in the *R. rickettsii* genome is consistent with this hypothesis.

## Introduction

*Rickettsia rickettsii*, a Gram negative obligate intracellular bacterium, is the causative agent of Rocky Mountain spotted fever (RMSF). In the United States, Rocky Mountain spotted fever is the most severe and frequently reported rickettsial illness [1]. Despite the effectiveness of early antibiotic treatment, approximately 1% to 10% of individuals who become ill with Rocky Mountain spotted fever die from the infection [2]–[4].

Various species of ticks are considered natural hosts of *R. rickettsii*. In the Rocky Mountain region, the Rocky Mountain wood tick, *Dermacentor andersoni*, is the major reservoir for *R. rickettsii*. In the eastern and southern United States, *R. rickettsii* is found in *D. variabilis*, the common American dog tick [5], [6]. Once *R. rickettsii* infects a tick it can persist for the duration of the tick's life cycle (larva, nymph, and adult) and be passed on to the next generation through transovarial transmission. Ticks can also become horizontally infected by feeding on infected mammals [5]–[9]. Transmission of *R. rickettsii* from the tick to the human host begins with attachment of the adult tick, the only developmental stage that feeds on humans. Pathogenic bacteria that cycle between environmental sources and mammalian hosts as part of their natural history modify their transcriptional profiles to adapt to their environments. In response to such changes in environments, bacteria typically employ global regulatory systems that sense environmental stimuli and modify gene expression profiles appropriately. Pathogens generally upregulate virulence genes only when in association with a mammalian host and temperature is an important signal regulating virulence gene expression. In addition to temperature, a number of factors including ion concentration, nutrient availability, pH, iron level, oxygen tension, osmolarity, and growth phase may also modulate gene expression [10].

*R. rickettsii* held in ticks for an extended time at low temperatures (to simulate overwintering) is believed to enter an avirulent state. If such ticks are permitted a blood meal, the rickettsiae subsequently revert to a virulent state in a process termed “reactivation” [6], [11], [12]. Reactivation of rickettsiae is thought to be induced by an increase in temperature, an influx of nutrients, or a combination of both. Once the tick initiates feeding, transmission of rickettsiae into the human skin occurs from the salivary glands of the tick. Rickettsiae inoculated into the skin spread via the lymphatic system and blood stream to all parts of the body. Damage to vascular endothelial cells by *R. rickettsii* leads to increased vascular permeability and leakage of fluid into the interstices causing the characteristic rash observed in RMSF [5], [13].

The response of *R. rickettsii* to different environmental conditions encountered during its transition from arthropod to mammalian hosts has not been fully investigated. To determine the capacity of *R. rickettsii* to respond to changes in environmental conditions, custom Affymetrix GeneChips were used to determine transcriptional changes in *R. rickettsii* exposed to differences in temperature, iron availability, cell type, and cold shock.

## Results

### Transcriptional response of *R. rickettsii* grown at 37°C or 34°C and 25°C or 22°C during early stationary and log phases of growth

**Figure 1:**
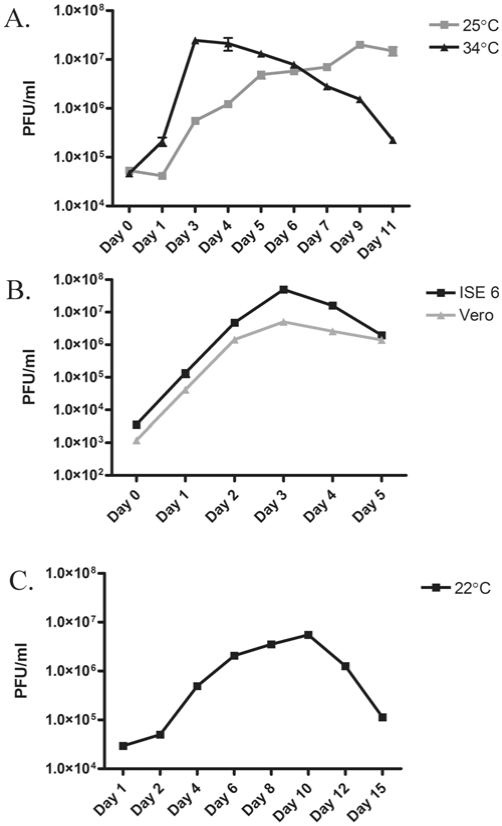
Growth kinetics of *R. rickettsii* under different experimental conditions. A) Growth of *R. rickettsii* in Vero cells incubated at either 34°C or 25°C. B) Growth of *R. rickettsii* at 37°C in ISE6 and Vero cells. C) Growth of *R. rickettsii* in ISE6 cells at 22°C.

**Figure 2:**
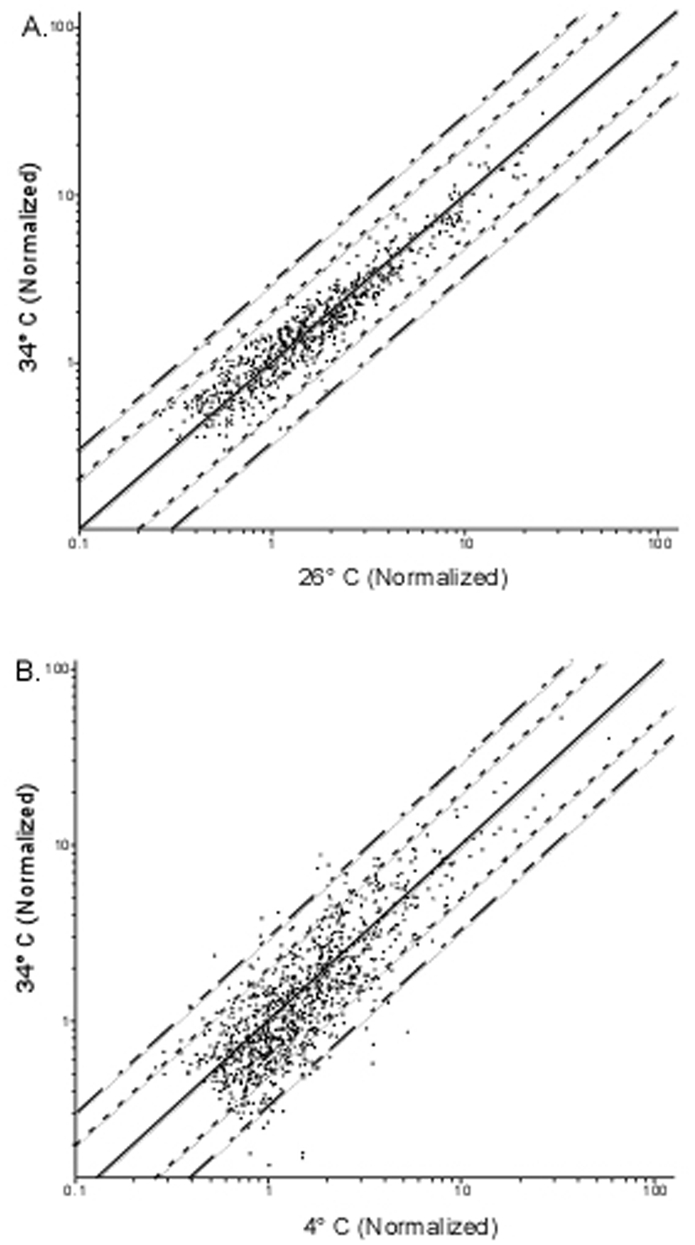
Scatter plots of fold changes. A) 34°C vs 25°C. B) 34°C vs 4°C. The solid line indicates an equivalence between the two conditions, while the small dashed line indicates a two fold difference and the long-short dashed line indicates a three fold difference.

*R. rickettsii* is subject to significant changes in temperature during growth within an infected tick depending on the ticks association with a mammalian host and feeding state. It has been hypothesized that growth of *R. rickettsii* at elevated temperature (during feeding) may induce a unique set of genes required for virulence [11], [12], [14]. To investigate transcriptional changes induced by growth temperature, *R. rickettsii* was grown in Vero cells incubated at 34°C or 25°C and in ISE6 cells incubated at 37°C or 22°C. *R. rickettsii* was harvested at late log phase or early stationary phase of growth (day two for late log phase and day three for stationary phase cultures grown at 34°C and day six for late log phase and day eight for stationary phase cultures grown at 25°C) (Fig. 1 A). Total RNA was then extracted and hybridized to custom Affymetrix GeneChips as described in the materials and methods. Affymetrix GeneChip (no. 3 RMLchip3a520351F) contains probe sets from both the virulent *R. rickettsii* strain Sheila Smith (CP000848) and avirulent *R. rickettsii* Iowa (CP000766). Comparison of *R. rickettsii* grown to late log phase or early stationary phase in Vero cells at 34°C or 25°C showed no significant changes in gene expression ≥3-fold (Fig. 2 A). Similarly *R. rickettsii* grown in ISE6 cells at temperatures of 37°C and 22°C to early stationary phase did not result in significant differences in gene expression ≥3-fold. A 3-fold cut-off was chosen as a conservative estimate of biologically significant changes in gene expression. These results indicate that *R. rickettsii* does not dramatically alter its transcriptional profiles in response to changes in growth temperature typically used in the laboratory to mimic environmental changes.

### Cold shock response of *R. rickettsii*

*R. rickettsii* is able to survive exposure to low temperature (4°C) for extended periods of time, equivalent to overwintering in infected ticks, while still remaining virulent upon gaining access to a mammalian host [11], [12]. To investigate the transcriptional response of *R. rickettsii* exposed to 4°C, *R. rickettsii* was grown for three days at 34°C in Vero cells, after which time the cultures were shifted to 4°C for 2 hrs and then transferred back to 34°C for an additional 2 hrs. At each of the temperature shifts, RNA was extracted and used to compare to RNA extracted from *R. rickettsii* grown for three days continuously at 34°C. Comparison of *R. rickettsii* maintained at 34°C for three days to *R. rickettsii* grown at 34°C for three days and then shifted to 4°C for 2 hrs revealed a total of 28 genes that were up or down regulated at least 3-fold. Sixteen of these genes were up regulated upon shift to 4°C while 12 were down regulated (Fig. 2 B). To confirm these changes, total RNA from infected cultures grown for three days at 34°C and then shifted to 4°C for 2 hrs was compared to total RNA from rickettsiae grown for three days at 34°C, shifted to 4°C for 2 hrs, and then shifted back to 34°C for 2 hrs. All 29 genes that displayed regulation after shift from three days of growth at 34°C to 4°C for 2 hrs were restored to their original levels of expression after being shifted back to 34°C from 4°C. (Table 1). These results indicate that *R. rickettsii* is able to respond transcriptionally to changes in temperature but the temperature differential is greater than that typical of bacteria alternating between mammalian and arthropod hosts.

**Table 1:** Transcriptional response of *R. rickettsii* shifted from 34°C to 4°C to 34°C.

*R.r* Sheila Smith (*R.r* Iowa)^a^	4°C 2 hrs/Day 3 34°C^b^ (Fold Change)	34°C 2 hrs/4°C 2 hrs^c^ (Fold Change)
RrIowa0897^d^	8.54	−8.3
RrIowa1169^d^	6.03	−6.66
A1G_02980 (RrIowa0627)	5.76	−5.26
A1G_01610 (RrIowa0341)	5.70	−5.55
A1G_00030 (RrIowa0006)	4.46	−3.4
RrIowa1039^d^	3.63	−2.9
A1G_06435 (RrIowa1378)	3.58	−3.22
RrIowa0142^d^	3.57	−3.22
A1G_03075 (RrIowa0648)	3.36	−3.03
A1G_03390 (RrIowa0718)	3.34	−3.22
A1G_04585 (RrIowa0965)	3.17	−2.85
A1G_04585 (RrIowa1422)	3.13	−2.38
A1G_06950 (RrIowa1484)	3.08	−2.04
A1G_06080 (RrIowa1305)	3.05	−2.85
A1G_06725 (RrIowa1438)	3.03	−2.43
A1G_00805 (RrIowa0176)	3.00	−2.56
RrIowa1023^d^	−3.03	3.08
A1G_05325 (RrIowa1145)	−3.03	2.42
A1G_01335 (RrIowa0288)	−3.12	2.32
A1G_05320 (RrIowa1144)	−3.12	2.39
A1G_05505 (RrIowa1187)	−3.12	2.95
RrIowa0846^d^	−3.12	2.77
A1G_01045 (RrIowa0225)	−3.44	2.73
A1G_03530 (RrIowa0748)	−3.70	3.44
A1G_06470 (RrIowa1384)	−3.84	4.94
A1G_05445 (RrIowa1175)	−4.34	3.9
A1G_05480 (RrIowa1182)	−4.54	4.48
A1G_05085 (RrIowa1095)	−5.00	4.61

a locus_tags for *R. rickettsii* Sheila Smith (CP000848) and Iowa (CP000766).

b Comparison of gene expression levels from *R. rickettsii* grown for three days at 34°C and shifted to 4°C for 2 hrs to *R. rickettsii* grown for three days at 34°C.

c Comparison of gene expression levels from *R. rickettsii* grown for three days at 34°C shifted to 4°C for 2 hrs and then back to 34°C for 2 hrs to *R. rickettsii* grown for three days at 34°C and shifted to 4°C for 2 hrs.

d No anotation in *R. rickettsii* Sheila Smith (CP000848).

### Transcriptional response of *R. rickettsii* to prolonged exposure to 4°C

**Figure 3:**
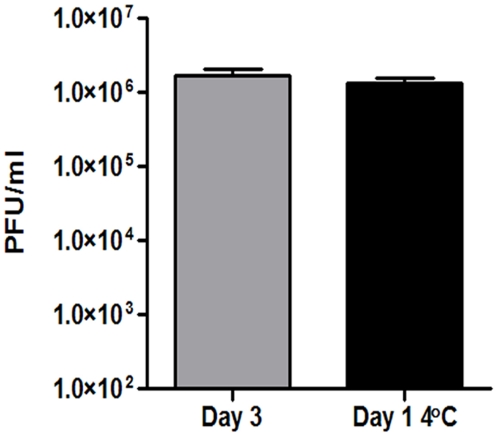
Survival of *R. rickettsii* after exposure to 4°C for 24 hrs. *R. rickettsii* grown for three days was shifted to 4°C for 24 hours after which PFUs were determined and compared to *R. rickettsii* grown for three days at 34°C.

To determine the gene expression profile of *R. rickettsii* after prolonged exposure to 4°C, RNA was also extracted from cultures incubated at 4°C for 24 hrs and after growth at 34°C for three days. Plaque forming units (PFUs) after incubation for 24 hrs at 4°C were similar to PFUs determined after growth at 34°C for three days (Fig. 3). Microarray analysis revealed a total of 56 genes regulated ≥3-fold, 49 genes were up regulated while 7 genes were down regulated (Table 2).

**Table 2:** Transcriptional response of *R. rickettsii* exposed to 4°C for 24 hrs.

*R.r* Sheila Smith (*R.r* Iowa)^a^	4°C 24 hrs/Day 3 34°C^b^ (Fold Change)
A1G_04305 (RrIowa0904)	18.55
A1G_04840 (RrIowa1040)	15.74
RrIowa1037^c^	14.29
A1G_04310 (RrIowa0905)	11.73
A1G_04835 (RrIowa1036)	10.82
RrIowa0890^c^	9.98
RrIowa1039^c^	9.18
A1G_00030 (RrIowa0006)	8.33
A1G_02195 (RrIowa0461)	7.37
A1G_07015 (RrIowa1497)	7.21
A1G_02820 (RrIowa0592)	7.04
A1G_01610 (RrIowa0341)	6.52
A1G_03075 (RrIowa0648)	6.21
A1G_00065 (RrIowa0015)	6.03
A1G_05945 (RrIowa1276)	6.00
A1G_01945 (RrIowa0410)	5.92
RrIowa0897^c^	5.75
A1G_06420 (RrIowa1375)	5.75
RrIowa1169^c^	5.64
A1G_05940 (RrIowa1275)	5.46
A1G_00285 (RrIowa0063)	4.73
A1G_05960 (RrIowa1280)	4.33
A1G_07355 (RrIowa1570)	4.26
A1G_06080 (RrIowa1305)	4.02
RrIowa0127^c^	3.86
A1G_06415 (RrIowa1374)	3.80
A1G_02825 (RrIowa0593)	3.76
A1G_07140 (RrIowa1526)	3.76
A1G_02575 (RrIowa0544)	3.75
A1G_01810 (RrIowa0384)	3.71
A1G_07135 (RrIowa1524)	3.69
A1G_06075 (RrIowa1304)	3.67
A1G_06375 (RrIowa1366)	3.61
A1G_00925 (RrIowa0201)	3.59
A1G_00120 (RrIowa0027)	3.52
A1G_03785 (RrIowa0796)	3.51
RrIowa1046^c^	3.49
A1G_07280 (RrIowa1556)	3.36
A1G_05420 (RrIowa1168)	3.34
A1G_06265 (RrIowa1346)	3.34
A1G_06435 (RrIowa1378)	3.30
A1G_01875 (RrIowa0396)	3.30
RrIowa0327^c^	3.29
A1G_04230 (RrIowa0891)	3.29
A1G_00070 (RrIowa0016)	3.25
A1G_03145 (RrIowa0663)	3.16
A1G_03190 (RrIowa0672)	3.15
A1G_05640 (RrIowa1217)	3.09
A1G_04585 (RrIowa0965)	3.08
A1G_01045 (RrIowa0225)	−3.22
A1G_00185 (RrIowa0040)	−3.22
A1G_06520 (RrIowa1396)	−3.33
A1G_05320 (RrIowa1144)	−3.57
A1G_04395 (RrIowa0923)	−3.70
A1G_05325 (RrIowa1145)	−4.16
RrIowa1023^c^	−4.76

a locus_tags for *R. rickettsii* Sheila Smith (CP000848) and Iowa (CP000766).

b Comparison of gene expression levels from R. rickettsii grown for three days at 34°C and shifted to 4°C for 24 hrs to R. rickettsii grown for three days at 34°C.

c No anotation in *R. rickettsii* Sheila Smith (CP000848).

Comparison of the genes that were up- or downregulated after exposure to 4°C for 2 hrs to genes up- or down-regulated after 24 hrs at 4°C revealed that most genes showed the same trend whether incubated for 2 or 24 hr at 4°C (Table 3). A few were differentially regulated after 2 or 24 hr at 4°C (Table 4). Those genes regulated to a greater degree after 24 hr at 4°C could represent genes involved in an adaptive response to extended exposure to 4°C as opposed to genes that are more highly regulated after 2 hr at 4°C and may represent those genes immediately responding to cold shock.

**Table 3:** Confirmation and comparison of responses after 2 and 24 hrs at 4°C.

*R.r* Sheila Smith (*R.r* Iowa)^a^	Microarray		QuantiGene		Function
	4°C 2 hrs/Day 3 34°C^b^ (Fold Change)	4°C 24 hrs/Day 3 34°C^c^ (Fold Change)	4°C 2 hrs/Day 3 34°C^b^ (Fold Change)	4°C 24 hrs/Day 3 34°C^c^ (Fold Change)	
RrIowa0890^e^	10.63	9.98			Hypothetical
RrIowa0897^e^	8.54	5.75			Hypothetical^d^
RrIowa1037^e^	6.68	14.29			Hypothetical^d^
A1G_04840 (RrIowa1040)	6.26	15.74			Hypothetical^d^
RrIowa1169^e^	6.03	5.64			Hypothetical^d^
A1G_01610 (RrIowa0341)	5.70	6.52	12.7^b^	11.9±1.4	Hypothetical
A1G_04835 (RrIowa1036)	4.84	10.82			Hypothetical^d^
A1G_00030 (RrIowa0006)	4.46	8.33			PEMK-like protein
RrIowa1039^e^	3.63	9.18			cysteine methyltransferase^d^
A1G_06435 (RrIowa1378)	3.58	3.30	2.5±0.7	2.5±0.3	MFS superfamily
A1G_03075 (RrIowa0648)	3.36	6.21			FKBP-PPIase
A1G_04585 (RrIowa0965)	3.17	3.08	2.2±0.5	2.2±0.1	glycoprotease family protein
A1G_06080 (RrIowa1305)	3.05	4.02			external DNA uptake
A1G_00805 (RrIowa0176)	3.00	2.66			acetylglutamate kinase
A1G_07355 (RrIowa1570)	2.85	4.26			RelB protein
A1G_03190 (RrIowa0672)	2.83	3.15			Hypothetical^d^
A1G_05420 (RrIowa1168)	2.71	3.34			SpoT-like
A1G_07015 (RrIowa1497)	2.6	7.21			Hypothetical
A1G_07135 (RrIowa1524)	2.6	3.69			Hypothetical
A1G_06415 (RrIowa1374)	2.59	3.80			outer membrane protein^d^
A1G_05960 (RrIowa1280)	2.49	4.33			multidrug resistance transporter
A1G_02195 (RrIowa0461)	2.38	7.37			sodium/proton antiporter protein
A1G_01945 (RrIowa0410)	2.37	5.92			Hypothetical
A1G_02575 (RrIowa0544)	2.32	3.75			Hypothetical
A1G_06265 (RrIowa1346)	2.32	3.34			ADP,ATP carrier protein
A1G_02820 (RrIowa0592)	2.26	7.04	2.7±1.0	7.7±1.5	ABC substrate-binding protein
RrIowa1046^e^	2.19	3.49			Hypothetical
A1G_02825 (RrIowa0593)	2.11	3.76			ABC permease protein
RrIowa0327^e^	2.09	3.29			ABC transporter protein^d^
A1G_03145 (RrIowa0663)	2.07	3.16			endopeptidase
A1G_03785 (RrIowa0796)	2.05	3.51			ADP,ATP carrier protein
A1G_00185 (RrIowa0040)	−2.27	−3.22			Hypothetical
A1G_04395 (RrIowa0923)	−2.38	−3.70			LSU ribosomal protein L36P
A1G_05325 (RrIowa1145)	−3.03	−4.16			10 kDa chaperonin GROES
A1G_01335 (RrIowa0288)	−3.12	−2.08	−2±0.3	−2.3±0.2	Chaperone protein dnaK
A1G_05505 (RrIowa1187)	−3.12	−2.32			LSU ribosomal protein L14P
A1G_05320 (RrIowa1144)	−3.12	−3.57			60 kDa chaperonin GROEL
A1G_01045 (RrIowa0225)	−3.44	−3.22			Methyltransferase
A1G_03530 (RrIowa0748)	−3.70	−2.77	−2.7±0.4	−2.9^b^	Hypothetical
A1G_06470 (RrIowa1384)	−3.84	−2.56	−2.5±0.4	−2.3±0.2	Hypothetical
A1G_05445 (RrIowa1175)	−4.34	−2.22			SSU ribosomal protein S13P
A1G_05480 (RrIowa1182)	−4.54	−2.77	−2.5±0.5	−4.4±0.6	LSU ribosomal protein L6P
RrIowa1023^e^	−3.03	−4.76			Hypothetical^d^
A1G_05085 (RrIowa1095)	−5.00	−2.22	−3±1.4	−3.2±1.3	patatin-like protein

a locus_tags for *R. rickettsii* Sheila Smith (CP000848) and Iowa (CP000766).

b Comparison of gene expression levels from *R. rickettsii* grown for three days at 34°C and shifted to 4°C for 2 hrs to *R. rickettsii* grown for three days at 34°C.

c Comparison of gene expression levels from *R. rickettsii* grown for three days at 34°C and shifted to 4°C for 24 hrs to *R. rickettsii* grown for three days at 34°C.

d Gene fragments or predicted genes less than 10 kda.

e No anotation in *R. rickettsii* Sheila Smith (CP000848).

**Table 4:** Genes that differentially respond early or late after shift to 4°C.

*R.r* Sheila Smith (*R.r* Iowa)^a^	Microarray		QuantiGene		Function
	4°C 2 hrs/Day 3 34°C^b^ (Fold Change)	4°C 24 hrs/Day 3 34°C^c^ (Fold Change)	4°C 2 hrs/Day 3 34°C^b^ (Fold Change)	4°C 24 hr/Day 3 34°C^c^ (Fold Change)	
A1G_02980 (RrIowa0627)	5.76	1.91			Hypothetical^d^
RrIowa0142^e^	3.57	1.51			Hypothetical^d^
A1G_06650 (RrIowa1422)	3.13	1.44	2±0.2	4±2.5	Hypothetical
A1G_03390 (RrIowa0718)	3.34	1.81			cytochrome b561^d^
A1G_06950 (RrIowa1484)	3.08	1.16	1.7±0.3	1.2±0.2	Septum protein Maf
A1G_06725 (RrIowa1438)	3.03	1.71			heme exporter protein
RrIowa0846^e^	−3.12	−1.85			Hypothetical^d^
RrIowa0127^e^	1.95	3.86			Hypothetical^d^
A1G_04310 (RrIowa0905)	1.91	11.73			Hypothetical^d^
A1G_07140 (RrIowa1526)	1.85	3.76			Hypothetical
A1G_06375 (RrIowa1366)	1.79	3.61	1.4±0.1	3.5±0.3	Hypothetical
A1G_00070 (RrIowa0016)	1.68	3.25			Hypothetical^d^
A1G_07280 (RrIowa1556)	1.66	3.36			cytidylyltransferase
A1G_04305 (RrIowa0904)	1.58	18.55	1.7±0.4	14.3±2.6	Hypothetical^d^
A1G_06075 (RrIowa1304)	1.5	3.67	2±0.4	5.8±0.3	endopeptidase
A1G_00925 (RrIowa0201)	1.46	3.59			Nucleotidyltransferase^d^
A1G_04230 (RrIowa0891)	1.45	3.29			Hypothetical^d^
A1G_00120 (RrIowa0027)	1.35	3.52			Hypothetical^d^
A1G_01875 (RrIowa0396)	1.32	3.30			Hypothetical^d^
A1G_00065 (RrIowa0015)	1.28	6.03			Hypothetical^d^
A1G_05945 (RrIowa1276)	1.27	6.00			Hypothetical^d^
A1G_06420 (RrIowa1375)	1.25	5.75			Hypothetical^d^
A1G_05640 (RrIowa1217)	1.25	3.09			Hypothetical
A1G_01810 (RrIowa0384)	1.08	3.71			dipeptidase^d^
A1G_00285 (RrIowa0063)	−1.12	4.73			Hypothetical^d^
A1G_05940 (RrIowa1275)	−1.16	5.46	1.9±0.4	8.8±0.6	Hypothetical^d^
A1G_06520 (RrIowa1396)	−1.81	−3.33			Hypothetical

a locus_tags for *R. rickettsii* Sheila Smith (CP000848) and Iowa (CP000766).

b Comparison of gene expression levels from *R. rickettsii* grown for three days at 34°C and shifted to 4°C for 2 hrs to *R. rickettsii* grown for three days at 34°C.

c Comparison of gene expression levels from *R. rickettsii* grown for three days at 34°C and shifted to 4°C for 24 hrs to *R. rickettsii* grown for three days at 34°C.

d Gene fragments or predicted genes less than 10 kda.

e No anotation in *R. rickettsii* Sheila Smith (CP000848).

To confirm the validity of the microarray results, QuantiGene 2.0 (Panomics) was used to directly quantify mRNA from biologically replicated experiments as describe above and in materials and methods. Of the genes tested, 13 out of 15 displayed similar fold changes to the microarrays thus confirming that the microarray data were reliable (Tables 3/4).

### Transcriptional response of *R. rickettsii* exposed to limiting iron conditions

**Figure 4:**
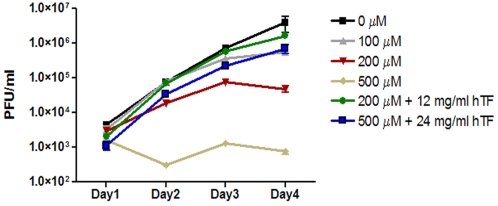
Growth of *R. rickettsii* under limiting iron conditions. A) *R. rickettsii* was grown on monolayers of Vero cells with increasing concentrations of deferozamine mesylate (0, 100, 200, and 500 µM) and PFUs were determined for days 1, 2, 3, and 4 after infection. To determine if the effects of high concentrations of deferozamine mesylate (200 and 500 µM) were reversible excess iron saturated holotransferrin (12 and 24 mg/ml respectively) was added to the media as an iron source.

During its life span, a tick molts twice; from a larva to a nymph and from a nymph to an adult. Ticks also go through periods when they are not feeding but seeking a new host (questing). During molting and questing periods *R. rickettsii* may encounter nutrient limiting conditions including iron limitation until the tick acquires another blood meal. Seasonal changes during the tick's life cycle could also present periods of temperature change and nutrient limitation. To further investigate the effects of limiting iron conditions, *R. rickettsii* was grown in the presence of concentrations of the iron chelating agent, deferozamine mesylate, known to inhibit growth of intracellular pathogens [15]–[17]. Titration of *R. rickettsii* growth in the presence of deferozamine mesylate indicated inhibition at concentrations of 200 and 500 µM (Fig. 4). However, growth of *R. rickettsii* was restored by the addition of excess iron saturated holotransferrin (12 and 24 mg/ml) to the media (Fig. 4). These experiments indicate that limiting the availability of iron to the rickettsiae inhibited their growth. To investigate the effect of limiting iron conditions on the transcriptional response of *R. rickettsii*, rickettsiae were grown for three days at 34°C before deferozamine mesylate (200 µM) was added to the media and incubation at 34°C was continued for 24 hours. Total RNA was extracted from rickettsiae at day three and 24 hrs after deferozamine mesylate treatment. Under these conditions, only 5 genes showed ≥3-fold change, with 3 genes up regulated and 2 down regulated under iron limiting conditions (Table 5). All of the genes were hypothetical proteins with no known function. Consequently their role in the response to iron limitation is unclear. These data indicate that *R. rickettsii* has a surprisingly limited transcriptional response to low iron levels under these experimental conditions.

**Table 5:** Transcriptional response to limiting iron conditions.

*R.r* Sheila Smith (*R.r* Iowa)^a^	Iron (−)/Iron (+)^b^ (Fold Change)	Function
A1G_00285 (RrIowa0063)	3.86	Hypothetical^c^
A1G_04310 (RrIowa0905)	3.83	Hypothetical^c^
A1G_04305 (RrIowa0904)	3.69	Hypothetical^c^
A1G_06120^d^	−3.03	Hypothetical
RrIowa1125^e^	−3.22	Hypothetical^c^

a locus_tags for R. rickettsii Sheila Smith (CP000848) and Iowa (CP000766).

b Comparison of gene expression levels from *R. rickettsii* grown for three days at 34°C and then incubated with 200 µM of deferozamine mesylate for 24 hrs to *R. rickettsii* grown for three days at 34°C.

c Gene fragment or predicted gene less than 10 Kda.

d No anotation in *R. rickettsii* Iowa (CP000766).

e No anotation in *R. rickettsii* Sheila Smith (CP000848).

### Transcriptional differences of *R. rickettsii* to growth in Vero or ISE6 cells

In the natural history of *R. rickettsii*, the organism occupies either arthropod or mammalian host cells. Host cell species can also influence pathogen gene expression patterns [18]. To determine if *R. rickettsii* displays differential gene expression dependent on host cell type *R. rickettsii* was grown in Vero cells at 37°C and ISE6 cells at 37°C or 22°C. *R. rickettsii* displayed similar growth kinetics in both cell lines (Fig. 2A,B,C) and RNA was harvested three days after infection at 37°C and eight days at 22°C. To determine differences in gene expression between cells types two comparisons were performed, Vero cells incubated at 37°C were compared to both ISE6 cells incubated at 37°C and 22°C. Microarray results showed 7 genes that were significantly changed ≥3-fold in at least one of the two comparisons (Table 6). However, none of these proteins have homologs with known function and their roles remain unknown. These results suggest that there are a limited number of *R. rickettsii* gene expression differences induced by growth in Vero cells opposed to ISE6 cells.

**Table 6:** Transcriptional differences between Vero and ISE6 cells.

*R.r* Sheila Smith (*R.r* Iowa)^a^	V37°C/I22°C^b^ (Fold Change)	V37°C/I37°C^c^ (Fold Change)	Function
A1G_01170 (RrIowa0254)	3.3	2.8	Hypothetical
A1G_02495 (RrIowa0526)	3.3	2.3	Hypothetical
A1G_05165 (RrIowa1113)	3.2	2.7	Hypothetical
A1G_05730 (RrIowa1234)	2.8	3.2	Hypothetical^d^
A1G_00870 (RrIowa0190)	−6.4	−3.1	Hypothetical^d^
A1G_07345 (RrIowa1569)	−4.4	−4.7	Beta-lactamase^d^
A1G_07140 (RrIowa1526)	−4.3	−3.9	Hypothetical

a locus_tag for *R. rickettsii* Sheila Smith (CP000848) and Iowa (CP000766).

b Comparison of gene expression levels from *R. rickettsii* grown for three days at 37°C in Vero cells to *R. rickettsii* grown for eight days at 22°C in ISE6 cells.

c Comparison of gene expression levels from *R. rickettsii* grown for three days at 37°C in Vero cell to *R. rickettsii* grown for three days at 37°C in ISE6 cells.

d Gene fragments or predicted genes less than 10 Kda.

## Discussion

During its life cycle *R. rickettsii* encounters several environmental challenges including nutrient availability, host cell type and growth temperature. To examine the global transcriptional response of *R. rickettsii* exposed to these different conditions, microarrays were used to determine transcriptional changes in response to changes in iron availability, host cell species and variations in temperature. Unlike other pathogenic bacteria that infect both arthropod and mammalian hosts, *R. rickettsii* exhibits remarkably little change in its gene expression patterns under conditions of changes in growth temperature, iron limitation, or host cell species. However, when exposed to more extreme shifts in temperature between 34°C and 4°C several genes respond ≥3-fold thus rickettsiae retain some ability to respond to environmental signals.

Many pathogenic bacteria respond to an increase in temperature to that of mammalian body temperature by expression of genes that promote survival in the mammalian host [10]. Typically, these pathogens occupy at some stage of their life cycle an environmental reservoir such as soil, water, or an arthropod host from which mammalian hosts become infected. Bacteria known to upregulate virulence gene expression in response to a shift to mammalian body temperature include *Yersinia*, *Borrelia*, *Leptospira*, *Franciscella*, *Shigella*, *Bordetella*, and *Streptococcus* [19]–[27]. In the laboratory, virulence gene expression is observed after a shift from moderate temperatures, 23–30°C to 37°C. *Yersinia pestis*, for example, upregulates many genes thought to be involved in virulence including the *ysa* type III secretion system. Indeed, the entire physiology of *Y. pestis* shifts at 37°C as the organism becomes autotrophic for many amino acids that it is capable of synthesizing at 26°C [25], [27]. Another arthropod borne pathogen *Borrellia burgdorferi* also regulates genes involved in pathogenesis in response to temperature shifts, including the surface protein OspC, which is expressed in mammals, and OspA, which is expressed in ticks [26], [28]. In contrast, *R. rickettsii* shows only a minimal response to temperature shifts from 22–25°C to 34–37°C with no genes up- or down-regulated ≥3-fold. A recent study of the closely related *Rickettsia typhi*, the flea-borne agent of murine typhus, also indicated a nominal response to shift in temperature from 25°Cto 37°C, with only two genes regulated ≥3-fold [29]. Because overwintering *R. rickettsii*-infected ticks are likely exposed to much greater temperature differentials, we repeated the genomic transcriptional profiling after maintaining infected Vero cell cultures for 2 or 24 hrs at 4°C to determine if this greater temperature shift was sufficient to globally alter gene expression. Under these conditions, 56 genes were differentially regulated ≥3-fold. *R. rickettsii* is therefore able to respond to environmental changes by modifying gene expression patterns but the temperature shifts required are greater than those typically used in the laboratory to induce virulence gene expression in other pathogens. The 4°C to 34°C temperature range examined here approximates the maximal temperature differentials expected to be encountered by rickettsiae in their natural environment. Ticks are killed by sub-freezing temperatures and survive overwintering by burrowing into forest debris to avoiding freezing, which would also be lethal to the rickettsiae [30]. Similarly the Rocky Mountain wood tick avoids the hot dry conditions of summer by insulating themselves within the soil.

Of the genes down regulated ≥3-fold upon shift from 34°C to 4°C were genes encoding three Major heat shock proteins (MHSP) GroES, GroEL and DnaK (A1G_05320/RrIowa1144, A1G_05325/RrIowa1145 and A1G_01335/RrIowa0288). MHSP are involved in the folding of newly synthesized proteins, preventing aggregation and repairing misfolded or damaged proteins [31]. In *R. conorii* and *R. felis* GroEL was reported as being constitutively expressed at high levels during mid-log phase of growth [32], [33]. Our results show that *R. rickettsii* down regulates GroEL under conditions that drastically slow the growth of *R. rickettsii*. In recent work investigating the starvation response in *R. conorii*, GroEL was also shown to be down regulated [34]. This suggests that under conditions of slow growth and stress, rickettsiae no longer require elevated expression of GroEL and subsequently down regulates its expression. In contrast, a number of MHSP including *groEL*, *groES* and *dnak* were upregulated ≥3-fold after exposure of *R. prowazekii* to heat shock [35]. Again, suggesting that under conditions of extreme stress ricketsiae are able to respond transcriptionaly.

Rickettsiae in both the spotted fever and typhus groups display phospholipase A_2_ (PLA_2_) activity [36]–[41]. After shift to 4°C *R. rickettsii* down regulates a gene encoding Patatin-like protein (A1G_05085/RrIowa1095), a storage glycoprotein found in high concentrations in potatoes and displays (PLA_2_) activity [42], [43]. The *R. rickettsii* Patatin-like protein has been predicted to contain PLA_2_ activity based on the observation that it contains the three residues identified as important for PLA_2_ activity [44]. It has been hypothesized that PLA_2_ activity plays a role in entry of the organism into host cells and subsequent escape of *R. rickettsii* from the early phagosome [36], [40].

In many bacteria, exposure to cold shock greatly induces the expression of cold shock genes within the *cspA* family [45]. *R. rickettsii* only contains one homolog of this family (A1G_05630/RrIowa1213) and its expression is only mildly up-regulated ∼1.7-fold in response to cold shock at 2 hours and ∼2.1-fold at 24 hours. However, a FK506-binding protein- type (FKBP) peptidylprolyl cis/trans isomerase (PPIase) (A1G_03075/RrIowa0648) was significantly upregulated >3-fold at both 2 and 24 hours. FKBP PPIases have been implicated in stress responses of other bacterial and are involved in protein folding and chaperon functions [46], [47].

*R. rickettsii* acquires many of the nutrients it requires for growth directly from the host cell cytoplasm [48]. Under conditions of low temperature many of these nutrients may become limiting as the metabolism of host cells slows. *R. rickettsii* appears to respond by up regulating many proteins with homology to transporters which are potentially involved in the transport of nutrients from the cytoplasm of host cells. The transcriptional response to nutrient limitation of a subset of genes in *R. conorii* has recently been published [34] and a number of homologs to those genes (GroEL, SpoT homologs, transporters, ADP/ATP carrier proteins, and integration host factor α (IHFα) were also found in our experiments regulated at least 2-fold. These findings suggest that incubation at 4°C may induce similar responses as exposure to nutrient limiting conditions.

Iron is an essential nutrient for most bacteria and under iron limiting conditions many bacteria respond by up regulating genes involved in iron scavenging [49]–[53]. Under iron limiting conditions, the growth of *R. rickettsii* is impaired and further reduction of available iron arrests growth. However, when microarrays were performed with RNA extracted from *R. rickettsii* exposed to iron limiting conditions and compared to RNA from *R. rickettsii* grown under normal iron conditions only four genes were found to be regulated in response to these conditions. This could be due to the fact that *R. rickettsii* may not normally encounter drastic changes in iron concentrations within its intracellular niche and has lost the ability to respond to limiting amounts of iron. One line of evidence to support this hypothesis is the lack of transcriptional regulators, including Fur, in *R. rickettsii* strains Iowa [54] or Sheila Smith (GenBank accession no. CP000848) that would classically be involved in gene regulation under limiting iron conditions. Interestingly, another obligate intracellular pathogen, *Coxiella burnetii*, that occupies a phagolysosomal-like vacuole [55], also exhibits a minimal capacity to respond to iron availability [17].

In tissue culture, *R. rickettsii* grows similarly in both tick and mammalian cells regardless of growth temperature. Microarray results indicate that under the conditions tested, there are minimal differences in gene expression ≥3-fold (7 genes) when comparing *R. rickettsii* grown in ISE6 cells to *R. rickettsii* grown in Vero cells. This could indicate that *R. rickettsii* does not regulate genes specifically for growth within mammalian or tick cells but contains a conserved set of genes that are required for growth in both mammalian and tick cells. This is different than the response of *Anaplasma marginale* to growth in arthropod vs. mammalian cell lines [18] but would be consistent with the hypothesis that *R. rickettsii* is reducing the size of its genome through reductive evolution to a core set of proteins that are required for survival within its intracellular environment independent of host cell species.

An analysis of potential transcriptional regulators within the *R. rickettsii* genome produced a surprisingly small number of predicted transcriptional regulators. These include a protein (A1G_05630/RrIowa1213) containing the conserved cold shock DNA-binding domain, the small histone-like protein HU (A1G_01205/RrIowa0261), intergration host factor IHFα/β (A1G_06050,03965/RrIowa1299,0832), the environmental sensor EnvZ (A1G_03330/RrIowa0706), and response regulator OmpR (A1G_03335/RrIowa0707), another environmental sensor NtrY (A1G_05210/RrIowa1124), and response regulators NtrX (A1G_04715/RrIowa1010) and CtrA (A1G_00620/RrIowa0131), along with the Helix-turn-Helix motif-containing proteins (A1G_03795,04220,06540,06700/RrIowa0798,0888,1400,1433), a CarD-like protein (A1G_00190/RrIowa_0041), DnaA (A1G_05055/RrIowa_1088), an AlgH homolog (A1G_00270/RrIowa_0061), and two Rrf2 family proteins (A1G_04135,04620/RrIowa_0870,0973). In contrast it has been predicted that *Escherichia coli* K-12 contains 314 regulatory DNA-binding proteins [56]. The small number of regulatory proteins contained within the *R. rickettsii* genome is consistent with the apparent lack of gene regulation under conditions that in other bacteria produce a number of transcriptional changes. None of these potential regulatory proteins displayed ≥3-fold changes in expression levels under any of the conditions examined.

The minimal transcriptional response to several environmental changes experienced by *R. rickettsii* in its life cycle is somewhat surprising. Rickettsiae may respond to unknown environmental signals other than those examined here. However, three well-known environmental factors, temperature, iron availability, and host cell type, known to upregulate virulence gene expression in other pathogenic bacteria induced only minimal changes in gene expression in rickettsiae. The genomes of rickettsiae are small (∼1.2 Mbp) and are believed to be undergoing reductive evolution [57]. The cytoplasmic compartment they inhabit may be so stable and similar between arthropods and mammals that rickettsiae do not experience threatening changes in enviroment and may have lost a measure of ability to respond to environmental changes. They are not totally unable to respond to environmental changes as 56 genes were up- or down-regulated more than 3-fold in response to more drastic shifts in temperature, although this value represents only about 0.04% of the *R. rickettsii* genome. The obligate intracellular habitat of rickettsiae may offer such a stable environment that the necessity of environmental gene regulation is much less than that of facultative pathogens.

## Materials and Methods

### Rickettsiae

*R. rickettsii* R strain was propagated in Vero cells with M199 medium or ISE6 tick cells [58] with L-15B medium and purified by Renografin density gradient centrifugation [59].

### Growth curves

To determine the growth rate of *R. rickettsii* R strain grown at different temperatures, *R. rickettsii* was grown on monolayers of either ISE6 or Vero cells in T25 flasks (MOI of 0.025). For the growth rates of *R. rickettsii* R strain under limiting iron conditions, rickettsiae were grown on monolayers of Vero cells (MOI of 0.025) in 6 well plates with 0, 100, 200 and 500 µM concentrations of deferozamine mesylate (Sigma). To restore growth in the presence of 200 or 500 µM deferozamine mesylate, iron saturated holotransferrin (Sigma) was included at concentrations of 12 and 24 mg/ml respectively. At the appropriate times after infection, the medium was removed and 1 ml of K36 (0.1 M KCl, 0.015 M NaCl, 0.05 M K_2_HPO_4_, 0.05 M KH_2_PO_4_, pH 7.0) was added. The monolayer was removed by scraping and the host cells were lysed by bead beating 2× for 4 seconds with (1 mm) glass beads. The cell suspension was then frozen at −80°C until plaque assays were performed. Plaque assays were performed as previously described [60]. All growth curves are representative of multiple experiments and each point is an average of at least two replicate platings.

### Microarray analysis

To investigate the transcriptional response of *R. rickettsii* exposed to limiting iron conditions, monolayers of Vero cells in T25 tissue culture flasks were infected with *R. rickettsii* R strain at an MOI of 0.025. To mimic iron limitation *R. rickettsii* were grown for three days at 34°C at which time 200 µM of deferozamine mesylate was added to the flasks and incubated for 24 hours. Total RNA was extracted by removing the growth media and adding 1 ml of Trizol (Invitrogen) to thee T25 tissue culture flasks grown for three days and to three T25 tissue culture flasks exposed to iron limiting conditions for 24 hrs.

To examine gene expression at different growth temperatures monolayers of Vero or ISE6 cells in T25 tissue culture flasks were infected with *R. rickettsii* R strain at an MOI of 0.025. Total RNA was extracted as above from five T25 tissue culture flasks grown at 37°C or 34°C for two and three days after infection and total RNA from five T25 tissue culture flasks grown at 25°C or 22°C for six and eight days following infection.

To study cold shock response in *R. rickettsii* 15 T25 flasks were infected with an MOI of 0.025 and incubated at 34°C for three days. After three days of growth five flasks were removed for isolation of total RNA and the remainder were shifted to 4°C for 2 hrs. After 2 hrs, total RNA from 5 flasks was extracted and the remaining five flasks were shifted back to 34°C for 2 hrs before total RNA was extracted. In a separate experiment six T25 tissue culture flasks were infected with an MOI of 0.025 and grown for three days at 34°C. As above total RNA was extracted from three T25 tissue culture flasks on day three after infection and three T25 tissue culture flasks were moved to 4°C for 24 hrs before extraction.

For iron limitation, growth temperature and cold shock plaque forming units (PFUs) were determined to ensure equal numbers of rickettsiae were being compared in each experiment. To determine non-specific background from ISE6 and Vero RNA, RNA was harvested from uninfected samples. These experiments showed a false positive rate of 2 to 5%. These genes were removed from the final analysis.

For GeneChip analysis RNA was harvested as previously described for all experiments [54]. Biotinylated *R. rickettsii* cDNA was hybridized to a custom Affymetrix GeneChip (no. 3 RMLchip3a520351F) containing 1,991 probe sets from two different *R. rickettsii* strains (Sheila Smith and Iowa). Hybridization was performed at 40°C for sixteen hours in a 500 k Affymetrix 640 oven. Each chip was stained with 2× MES buffer with 50 mg/ml BSA and 1 mg/ml streptavidin phycoerythrin. The stained GeneChips were scanned using the Affymetrix 7Gplus GeneChip scanner to create image files. GeneChip Operating Software (GCOS v1.4) (Affymetrix) was used to convert the image files to cell intensity data files. The data were normalized using the scaling method within GCOS with a scaled target of 1500 using a *R. rickettsii* probe set filter to produce analyzed files, report files and a pivot table. Quality control analysis using Affymetrix hybridization controls (Affy 900455) creX 100 pM, bioD 25 pM, bioC 5 pM, bioD 1.5 pM and poly-A RNA control stock produced the expected signal gradients. The pivot table was imported into GeneSpring GX 7.3 (http://www.chem.agilent.com) and used to create a hierarchical clustering using a Pearson correlation similarity measure with average linkage to ensure that replicates for each condition were clustering together. Similarly, data files were analyzed using Partek Genomics Suite software v6.3 to produce principal component graphs to determine if the replicates were clustering. In all cases both analyses showed that the replicates clustered. The data was analyzed after ANOVA was performed to produce false discovery rate corrected p-values (0.05) for each probe set and with strict criteria placed on p-value, fold change and quality (signal and call consistency). Only genes that were present in at least 4 out of 5 chips were included in the final analysis. The GeneChip data are posted on the Gene Expression Omnibus (GEO, www.ncbi.nlm.gov/geo/, accession number GSE14965).

### Quantigene 2.0

To determine the validity of our microarray results Quantigene 2.0 (Panomics) was used to measure levels of mRNA directly from infected cells. Infections were done in 24 well plates with an MOI of 0.025 under previously describe growth conditions and levels of mRNA were determined according to the manufacturer's instructions. A1G_05810/RrIowa1247 which displayed no change in expression under all growth conditions tested, was used to normalize the signal in Quantigene 2.0 experiments.

### Gene locus_tags

When possible, gene locus_tags were given for both *R. rickettsii* Sheila Smith (CP000848) and *R. rickettsii* Iowa (CP000766) genomes.
